# Revisiting Urban Street Planning and Design Factors to Promote Walking as a Physical Activity for Middle-Class Individuals with Metabolic Syndrome in Cairo, Egypt

**DOI:** 10.3390/ijerph21040402

**Published:** 2024-03-26

**Authors:** Hisham Abusaada, Abeer Elshater

**Affiliations:** 1 Housing and Building National Research Center, Giza 1770, Egypt; habusaada@yahoo.com; 2 Faculty of Engineering, Ain Shams University, Cairo 11517, Egypt

**Keywords:** public health, pedestrianization, planning and design practices, smart growth, socio-economic disparities, urban streets, walkability

## Abstract

This paper revisits the planning and design factors of “pedestrianized” and “walkable” urban streets to encourage physical activity, focusing on their prioritization according to public health and smart growth. The aim is to create a conceptual framework for urban planners and designers to encourage walking and reduce metabolic syndrome (MetS) risks. Through a scoping review, the study found that while pedestrianized and walkable streets share many planning and design factors, they have different objectives. The study explores how urban planning and design can reduce MetS risk among middle-class individuals using online video storytelling for 30 participants in three districts of Cairo, Egypt: El Zamalek, Old Cairo, and Heliopolis. It identifies three factors to address MetS symptoms for middle-class individuals: strategic, design-oriented, and technical. Practitioners and policymakers can use this framework to evaluate the impact of their work. This study is particularly relevant for cities in the Global South that are facing similar challenges.

## 1. Introduction

Metabolic syndrome (MetS), also known as insulin resistance or syndrome X, is a condition characterized by high blood sugar levels, low HDL cholesterol, blood pressure, triglycerides, high-density lipoprotein, and an “apple-shaped” body [1,2]. Lifestyle modifications can lead to various risk factors and emerging variables that contribute to the development of type 2 diabetes and cerebrocardiovascular disease [1,3,4,5].

A significant proportion of the urban population in the Global South developed MetS due to a sedentary lifestyle and unhealthy dietary habits [6]. In Egypt, in 2015, 55% of the population suffered from MetS, 85.6% of that number also had diabetes, and 76.6% among those had high blood pressure [3,7]. One reason for this high percentage was excess weight due to genetic variability [8], excess weight as a result of obesity [9] and lack of physical activity [10,11,12]. In all cases, doctors recommend a healthy diet and 150 min of walking per week, broken up into 30-min sessions five days a week [13,14,15], encouraging an active lifestyle [16] and a healthy environment that avoids sedentary behavior [17].

MetS symptoms can range from mild to severe and vary from person to person [18]. These symptoms may include emotional regulation and coping [19]; chronic kidney disease [20]; polyuria, polydipsia, polyphagia, weight loss, and blurred vision [18] diabetes fatigue dispositional mindfulness and depression [21]; eye diseases, including diabetic retinopathy, cataracts, macular degeneration, glaucoma, and dry eyes [22]; peripheral neuropathy [23], osteoporosis is a condition in which bones become weak and brittle and expose to falling and slipping risks [24] a diabetic foot ulcer, which is caused by hammertoe or Charcot arthropathy. Treatment includes walking with crutches and therapeutic sandals with soft, molded insoles, followed by adjusted or custom shoes [25]; anxiety: tension, a feeling of mental or emotional strain [26] hyperinsulinemia and inflammation that has been attributed to higher Body Mass Index (BMI) [27]; visceral fat tissue causes inflammation, which can lead to heart disease and stroke as white blood cells fight infection and damage to blood vessels [28] and psychological function, including mood and cognition, anxiety, depression, stress, and sadness [9].

Research on “pedestrianized” or “walkable” urban street planning and design practices does not always clearly distinguish between those that meet public health requirements with comfort and safety on the one hand and those that support walking as a physical activity to reduce MetS risks, mainly while considering social and economic differences in another aspect. Urban planning and design articles still lack a study clarifying which type is most suitable for middle-class individuals with MetS: “pedestrianized” or “walkable” urban streets. Moreover, the factors for planning and designing walkable urban streets as a physical activity from the perspective of their suitability for middle-class individuals with MetS are rarely applied or prioritized according to those patients’ preferences. The literature gap highlights the absence of studies in Egypt investigating how MetS influences walking as a physical activity among middle-class residents and its correlation with patterns and factors of urban street planning and design, including their prioritization.

In most Egyptian cities, urban planning prioritizes car mobility over pedestrian-friendly streets, leading to decreased walking activity and reliance on cars, exacerbating traffic congestion, CO_2_ emissions, and air pollution [29,30]. This lack of pedestrian-friendly infrastructure contributes to reduced physical activity, potentially leading to metabolic syndrome-related health issues [3]. Additionally, economic challenges faced by a significant portion of the population limit access to recreational activities and safe walking environments, further emphasizing the need for urban planning that prioritizes pedestrian safety and accessibility over car-centric designs [31,32].

In the past several decades, “walking” has played an essential role as a “physical activity” through the lenses of public health and smart growth. Physical activity encompasses various activities such as work-related tasks, leisure, sports, housework, cycling, and even just moving around. Studies by Frank, Kavage, and Litman [33], Brownson et al. [10], Gebel et al. [34], Ainsworth and Macera [35] and Kotecki [36] highlighted the close relationship between physical activity and public health. Also, the Center for Disease Control and Prevention (CDC) in 2022 [13] and the Department of Health in the Australian state of Victoria in 2023 [15] have also endorsed the benefits of physical activity.

Research conducted in journals specialized in behavioral medicine [34], preventive medicine [10], and transpiration and heath [37] has linked walkable neighborhoods with better health outcomes, higher physical activity levels, and overall improved health. Some research has focused on walking as a physical activity for older adults [24]. As shown by research published in journals such as *Sports* [2], the *Brazilian Journal of Sports Medicine* [14], and the *Journal of Physical Therapy Sciences* [38], walking as part of daily life experiences can significantly improve overall health and reduce the MetS risks.

For this study, it was interesting to investigate commonalities, fundamental differences, strengths (facilities) and weaknesses (barriers), and planning and design factors between pedestrianized and walkable urban streets. This study offers insight into the objectives and attributes of pedestrianization and walkable streets by conducting a scoping review based on content analysis. Furthermore, employed episodic narrative-based storytelling helps determine which of the two styles—pedestrianized or walkable—is favored by middle-class individuals with MetS from a health perspective, as suggested by medical professionals in their literature. In addition to addressing the challenges of the lack of safe and low-cost walking routes to improve public health, this study seeks to understand and prioritize urban planning and design factors in urban areas to mitigate this syndrome. The study will use participants’ stories as a basis for its findings.

A conceptual framework that urban planners and designers can utilize to ensure that streets are designed to suit the requirements of reducing MetS risks. To achieve this aim, the study sets three objectives. Revisits planning and design of two types of urban streets: “pedestrianized” or “walkable” to encourage walking as a physical activity. The study also examines which form of urban street is most conducive to physical activity and can help mitigate MetS risks: pedestrianized or walkable. 

The study’s significance is evident in its identification and prioritization of critical factors essential for planning and designing streets that promote physical activity, specifically targeting middle-class individuals with MetS in Cairo, Egypt. This framework provides valuable insights for researchers seeking to prioritize urban street planning and design elements that foster physical activity within this demographic. Moreover, practitioners and policymakers can leverage this framework to assess the effectiveness of their interventions, making it highly relevant to urban planning and public health initiatives.

## 2. Research Background

This study examines smart growth, pedestrianization, and walkability concepts, hypothesizing their potential impact on mitigating symptoms associated with MetS. The literature describes various paradigms of creating pedestrian spaces that prioritize safety, comfort, economic feasibility, and aesthetic appeal [39,40,41,42].

Smart growth research aims to understand how promoting accessible, fast, and direct walking can positively impact public health, particularly in urban environments, through transportation and land use policies and practices [43]. Studies have shown that incorporating regular walking into one’s lifestyle can have significant health benefits. These benefits include reducing chronic disease risk, improving cardiovascular health, and boosting mental well-being [33]. In addition, promoting walking can lower obesity rates and enhance academic performance [43]. It can also help older adults maintain physical function and independence to decrease the risk of falls [24] and improve the quality of life through urban trees [44] and healthy lifestyles by incorporating nature into healthcare strategies [45].

“Pedestrianized” or “walkable” urban streets are intended to improve the interaction between pedestrians and vehicles in cities, and each approach is different. Pedestrianized urban streets designate only one street for pedestrian use in an urban area that also accommodates automated traffic on other streets. In contrast, walkable urban streets are designed to facilitate walking on all urban streets to make daily activities more accessible and safer. From the perspective of participants, the narrative interview explores whether there is a need to reconsider the layout and design of two types of urban streets: “pedestrianized” or “walkable” to encourage walking as a physical activity, and which of the two styles is most suitable for middle-class individuals with MetS: pedestrianized or walkable urban streets. It also shows the extent to which planning, and design factors of both types are compatible. Their priority is arranged according to their desire to practice walking as a physical activity, not just for movement and mobility.

To improve the interaction between pedestrians and vehicles in cities, we expanded our research to focus on two common patterns of urban planning and design paradigms. Improving the interaction between pedestrians and cars in cities has been a popular strategy in urban planning and design literature for several decades. Since the 1920s, the term “pedestrianization” has been used to refer to the process of designating a specific place, street, community, or environment only for pedestrian use. This means all motor vehicles are excluded and separated from any pedestrian traffic [46,47,48].

The term “walkability” emerged in the second decade of the 2000s, which refers to the ease of pedestrian access to activities on the streets of residential neighborhoods and attractive destinations [41,49], safety, comfort, and overall enjoyment of walking around on foot [6,50,51]. Contemporary urban planning and design focus on the relationship between people and their surroundings. Walking has been increasingly promoted in recent years, and paradigms to encourage more people to walk to maximize the public health benefits of walking, such as New Urbanism [52,53] and smart growth [33,43,54]. Such trends include pedestrian-friendly streets [42,55,56], pedestrian-friendly environments or spaces [42,57], pedestrian-friendly communities [57] 10-min neighborhoods [58] 15-min cities [59], 20-min neighborhoods [60], x-minute cities [61,62], pedestrian-oriented [62], transit-oriented development [63] complete streets [55,59,64], and a short Perceived Walkability Scale [34,65,66].

The literature above highlights the crucial role of promoting accessible walking to enhance public health in urban areas through smart growth, pedestrianization, and walkability. Regular walking offers numerous health benefits, including reducing chronic disease risk and improving well-being. However, there is a need for more precise distinctions between pedestrian-friendly urban planning practices that prioritize public health and those specifically tailored to support walking as a physical activity to mitigate MetS risks, particularly among middle-class individuals. Future studies should address this gap by investigating how MetS affects walking behaviors and examining urban street planning factors catering to middle-class residents’ needs. Additionally, contemporary urban planning approaches emphasize pedestrian-friendly environments and communities, with strategies such as New Urbanism and smart growth aimed at promoting walking and maximizing its health benefits. 

## 3. Materials and Methods

### 3.1. Research Design and Data Collection

This study employed two qualitative approaches to gather comprehensive and descriptive data: a bibliometric study [67,68,69] and an ethnographic study [70]. The first approach of our bibliometric study consists of two consecutive techniques. We conducted a scoping review and content analysis using the first and second techniques. These techniques were used to identify commonalities, key differences, strengths (facilities), weaknesses (barriers), and planning and design factors for both types. These two techniques included three procedures as described. The first procedure of the first approach determined the scope of the investigation, with a focus on environmental and social sciences as the subject areas. It includes three categories: public health, medicine, psychology, nature landscape planning, geography, planning, development, urban studies, and transportation. Table 1 considered subject categories, the h-index, and the best quartile (Q1, 2, and 3) for 26 journals.

In the second procedure of the first approach, we determined a group of words about the proposed research, which could be found using the back-and-forth snowball technique after a complete reading of the previously available publications dealing with the research topic. These words are pedestrianization OR pedestrianized streets, AND walkability OR walkable streets, AND pedestrian streets OR pedestrian areas OR pedestrian-only, AND physical activity OR healthy communities. For this purpose, we selected 38 peer-reviewed articles in English from various sources. We utilized the SCImago Journal Country Rank (SJR) to rank these journals. Based on the Scopus database, a detailed summary of articles from 2004 to 2024 can be found in Table A1A in Supplementary Materials. We have excluded grey literature, such as book series, conference proceedings, and trade journals. We have not searched for other databases like Web of Science or Google Scholar.

During the third procedure of the first approach, we employed a content analysis technique to evaluate and determine which sources of information were included or excluded. This analytical process involved examining and interpreting the contents of each source to determine if it met the study’s relevance, reliability, and validity criteria. By using this method, we ensured that only the most appropriate and trustworthy sources were included in the study, which ultimately enhanced the quality and accuracy of our findings.

The second approach is ethnographic fieldwork using an episodic narrative-based storytelling technique [71,72,73]. This technique attempted to explore a socio-economic analysis and a storytelling technique based on episodic narration to examine the experiences of middle-class individuals with MetS with walking as a physical activity.

The second approach employed ethnographic techniques within a case study framework [74]. Throughout the second approach and its technique, this study used four procedures to come after the procedures of the first approach to gain a better understanding of the views expressed in a case study context. In the fourth procedure of the second approach, we collected contextual information from over a hundred men and women aged between seventy and ninety. These individuals belonged to the middle class and had advanced degrees. Our focus was on their scientific knowledge and awareness of MetS, which includes high blood pressure, type 2 diabetes, and triglycerides, among other associated conditions. Our investigation was not limited to obesity, as the disease could occur without it.

We selected 40 people who met the conditions of the fifth procedure of the second approach. We excluded those who did not have higher education since our questionnaire involved medical terminologies in the English language. It may have been challenging for those with less education to comprehend and articulate their experiences. We acknowledge that excluding less educated or illiterate groups is a limitation of our study, and we aspire to find ways to overcome this limitation in future research. We also acknowledge the potential bias in our sample, as higher education is linked with better income and health outcomes. Therefore, our findings may only be representative of some of the population.

**Figure 1:**
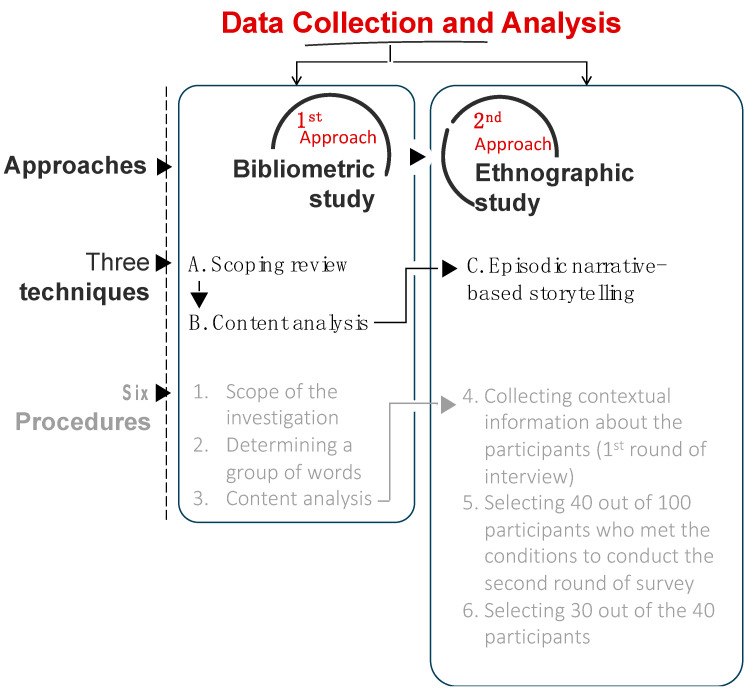
Research design.

In the sixth procedure of the second approach, a second round of review was conducted to narrow the number of participants to 30. Of these, 15 were in their sixties, 11 were in their seventies, and only four were in the octogenarian age group. It is important to note that these numbers do not represent the demographic composition of El Zamalek, Old Cairo, and Heliopolis districts. Additionally, all participants had at least a college education or higher. Figure 1 shows the data collection flowchart.

### 3.2. Case Study

In Egypt, urban decision-makers, planners, and designers focus on promoting car mobility and moving beyond environmentally friendly or green streets [29]. Walking in Cairo is becoming less popular and more complex in existing urban residential improvement projects and planning and designing new cities and communities [30]. As a result, cars are the primary mode of transportation in Cairo, leading to traffic congestion [75], high CO_2_ emissions, and increased air pollution [76].

Therefore, a significant challenge facing pedestrians in residential neighborhoods or newly developed urban communities in Cairo, Egypt, is the need for pedestrianized or walkable urban street planning and design practices to consider walking as both a mode of transportation and physical activity. This leads to decreased physical activity, which can cause MetS, including type 2 diabetes and cardiology [4]. The issue of walking in Egypt needs to be addressed to promote a healthier and more sustainable lifestyle. Regardless, no official statistics regarding the percentage of patients who suffer from MetS [4].

The other major problem in Egypt is latent, as 78% of the population belongs to the poor and vulnerable groups and the lower middle-income class [31]. Due to the fluctuating economic growth in Egypt, this class has faced significant hardships [32], which has led to a noticeable decline in the income of many families [77]. Consequently, this led to their loss of financial ability to enjoy recreational activities, as they had memberships in private clubs, paid-for housing in gated residential compounds, or access to gated gardens and waterfronts exclusive to the elite. Unsafe sidewalks and traffic limit outings [72,78,79]. Malls have better walking environments but are crowded and challenging for older adults and those with walking difficulties [72,80].

This study adopted a case study approach [74]. This research conducted an ethnographic study using episodic narrative interviews in three distinct districts of Cairo: Zamalek, Old Cairo, and Heliopolis. These districts were chosen because they represent a diverse mix of individuals from various socioeconomic backgrounds, including those who recently experienced rapid economic growth. This makes them an excellent location for investigating socioeconomics in Cairo, particularly observing the shift from the wealthy and upper-middle class to the low-income and lower-middle class. The team also factored in the walkability of the urban streets since the three districts were designed in the early beginnings to have walkable streets; the center of Old Cairo today also has pedestrianized streets.

Moreover, the chosen areas offer a diverse blend of people from different socioeconomic groups, making them an ideal location for sampling the middle class. We have selected Cairo as our focus city because we understand pedestrians’ challenges. It is important to note that certain factors relevant to Cairo may only apply to some cities in the Global South and may be seen as evident and unnecessary in some Global North cities. Due to the economic crisis, some middle-class individuals need help to afford commercial and recreational activities. In addition, despite many urban street sidewalks being closed for their extension. Some urban streets are only accessible from specific entry points, creating conflict because they are meant to be public. While local authorities manage this issue in residential areas, in some cases, the owners of large projects consider the sidewalks and surrounding roads private property rather than public.

### 3.3. Participants

The sample was mainly composed of middle-class individuals whose financial abilities were affected by the economic crisis and who could not afford to join social clubs, walled gardens, and gated communities. The final sample size was selected based on the criteria of having at least one family member with MetS, having a high level of education, and willingness to participate in the study. Fifteen men and fifteen women participated in the discussion, ensuring equal representation for both genders while considering their ages and fields of study. We ignored their original residence and thought that each sample member had a connection to the three study areas and that he had previously visited and walked in them for long periods. All participants confirmed their intimate knowledge of those areas to the point that they were the favorite areas to visit at different times.

We focused on two conditions of MetS to filter the final number of participants in our narrative study. Firstly, we assessed the scientific knowledge and awareness of the participants regarding MetS and whether they suffered from any of its associated diseases, such as high blood pressure, type 2 diabetes, and triglycerides. Research did not focus solely on obesity, as we concluded that the disease may not be present in all cases. Secondly, we evaluated the spatial knowledge of the participants to get a solid understanding of the three studied residential districts. We also walked through them before starting the interviews. The study focused on three age groups: 60s, 70s, and octogenarian (between 80 and 89 years old) and analyzed the characteristics of the participants in each group.

Table 2 included the participants’ profiles, illustrating the age group, gender, number of participants, job specialization, residence place, and MetS symptoms for each participant. We conducted three open-ended interviews using the online video call during our ethnographic study. The interviews were conducted in Arabic on weekends between November and December 2023. Each interview lasted between one and two hours. We asked the participants to speak freely about their experience walking in the pedestrianized and walkable streets in the three areas.

Specifically, we asked them to share their challenges as those who need to walk for half an hour. The back-and-forth focused on only three topics and aimed to gather insights from the experiences of Cairenes that can assist urban planners and designers in creating more walkable streets for middle-class individuals with MetS. These questions include:

- Is it necessary to have urban streets that encourage walking as a physical activity?
- Which urban street pattern is more suitable: pedestrianized or walkable?
- What factors should be added, and in what order?

These discussions led to the selection of a set of narratives to identify the type most suitable for middle-class individuals with MetS in three districts in Cairo and to identify some urban street planning and design practices. We derived many factors from the participants’ narratives and noticed that there are factors of utmost importance, weak importance, and moderate importance. However, after we finished listening to the narratives, we extracted the factors and presented them in a list to the participants. Then, we asked them to rank them in order of priority.

Upon analyzing the stories mentioned earlier, it was observed that critical findings emerged from three distinct categories of factors. Each participant was asked to rate each item mentioned in the stories on a scale of 1 to 10, with 10 being the highest score. After scoring each item, the participants were asked to determine the prioritization of the items based on the scores they assigned. This process allowed for a deeper understanding of the factors that influenced the outcomes of the stories.

## 4. Results

The following section presents two sets of research findings. The first set is based on a scoping review and focuses on content analysis data related to pedestrianization and walkable paradigms. It identifies commonalities, fundamental differences, strengths (facilities), weaknesses (barriers), and planning and design factors of each type of urban street. The second set of findings is based on insights collected through storytelling and episodic narrative interview techniques during online video calls. These insights are based on the opinions of a selected sample regarding their use of walking to manage metabolic syndrome, their preferences for pedestrianized or walkable streets, and urban street planning and design factors and their priority among middle-class individuals with metabolic syndrome in Cairo, Egypt.

### 4.1. Results of the Scoping Review

The results of the context review focus on two conceptual and empirical classifications to discuss enhancing interaction between pedestrians and vehicles in cities. The scoping review aims to objectively compare pedestrianized and walkable urban streets, focusing on four aspects: extracting commonalities, basic differences, strengths (facilities) and weaknesses (barriers), and planning and design factors.

#### 4.1.1. Extracting Commonalities

The first classification is pedestrianization, which involves creating a space entirely closed to vehicles, allowing pedestrians to move freely [40,64,81]. The second classification is walkability, which ensures that pedestrians can comfortably, safely, and quickly move between different city activities within specific walking distances, even in the presence of other modes of transportation [10,56]. A walkable environment promotes livability, comfort, and a pedestrian lifestyle in a city [56]. Perceived walkability is linked with the distance older people are willing to walk, walking time, and functional mobility [65,66]. 

These two approaches have different terms or concepts from each other. The scoping review showed that the first taxonomy includes terms and concepts, e.g., pedestrians only [46,81] pedestrian zone movement, pedestrian malls, a complete street, pedestrianized streets, and pedestrian corridors [40,57]. The second includes terms and concepts, e.g., walkable city [6,40] walkable environments [40], walkable neighborhoods [10,82] walkable communities, walkable streets or walkable urban streets [17,62,82], and urban walkability or street walkability [17]. A review of the current literature shows that classifications of pedestrianization and walkability may follow a transit-oriented development strategy [63]. Furthermore, the concept of walkability follows the “idea of a “street for all” and is close to the specifications of the American reformer “the complete street” [55].

Regarding the commonalities between these two patterns of streets, according to the literature review, both typologies follow the five physical characteristics of urban streets by urban design: imageability, enclosure, human scale, transparency, and complexity [83,84]. Both patterns also have crosswalks and traffic signals that help crossroads safely, which is extremely important to pedestrians [84,85]. Pedestrianized and walkable urban streets share similar features that enhance the quality of walking conditions, such as health, safety, comfort, security, mobility, pollution reduction, and cleanliness [85]. In addition, when planning pedestrian facilities, it is essential to consider suitable pavements, as pedestrians tend to walk where they feel comfortable [86].

#### 4.1.2. Basic Differences

The study results show a significant difference between “pedestrianized” and “walkable” urban streets. The former pattern repurposes the street space from automobile to pedestrian use, separating vehicle and pedestrian traffic [40]. It allows vehicular deliveries only during specific hours and relies heavily on public transportation. This concept differs from a walkable street, which aims to enable people to reach their attractive destinations on foot without relying on cars or public transportation [41,87]. To achieve this, neighborhoods should have safe, secure, accessible, comfortable, and attractive streets supported by appropriate policies [41,88].

Pedestrianization restricts streets to pedestrian use only to revamp outdoor shopping activities and improve the outdoor environment in the vicinity of this area. In contrast, the second mode improves walkability as a critical component of practical, accessible, equitable, sustainable, and livable communities [79]. Unlike the first approach, the second applies to all areas, not just commercial ones. Pedestrianization is effective in encouraging walking for amenity and walkability [40,42], and it is also a part of an economy for pedestrianizing the environment by influencing land-use changes and store density [42]. In this vein, the literature review did not show that walkability considers converting commercial streets to pedestrians.

The concept of pedestrianization, regarded as a catalyst for local economic development, should be thoroughly evaluated in conjunction with various design elements such as signage systems, barrier-free equipment, streetscape furniture, public arts, green spaces, roofs, and pavements. According to Murakami et al. [40] promoting local economic development is one of the strengths of walkable environments. They provide opportunities for local businesses to flourish and attract new investments. Walkable environments help households save money by reducing the need for private transportation and providing greater accessibility to essential services. On the other hand, streets planned and designed for walkability go beyond economic returns to achieve all other environmental incentives, not just local economic development.

Pedestrianized streets are streets where only pedestrians are allowed to walk, and no vehicles are permitted to enter. These streets are usually designed for pedestrians to walk around a specific destination. On the other hand, non-pedestrianized streets are those where pedestrians and vehicles share the same space, and pedestrians walk around multiple attractive destinations [41,87]. Because of this, on pedestrianized streets, the flow of pedestrians is determined by the destination’s attractiveness. In contrast, on non-pedestrianized streets, the flow of pedestrians is influenced by the street’s characteristics [49].

#### 4.1.3. Strengths and Weaknesses

Pedestrianization can help protect the vital functions of a town center from the negative impacts of traffic while still allowing access to motor vehicles. Redesigning urban streets to prioritize pedestrians can encourage social aspects of life while considering human relationships and behavior, such as controlling traffic, reducing traffic conflicts, and stimulating shopping [81]. Additionally, it can increase the speed and efficiency of pedestrian movement. It discourages car dependency and makes interaction and conflict between pedestrians and vehicles almost inevitable. In the same vein, walking is crucial as it reduces traffic, has low environmental impact, promotes social and recreational benefits, and enhances mental and physical health [89].

Moreover, prioritizing pedestrians is a measure to combat air, noise, and visual pollution in central areas [90]. It encourages people to walk and socialize, promotes tourism, and ensures the financial viability of inner-city retail stores. These stores are beginning to face serious competition from suburban shopping centers. However, car culture still dominates, resulting in a lack of commitment to pedestrians. This is evident in the numerous obstacles faced by the pedestrianization movement, including difficulty in cost recovery, issues with access to delivery vehicles, management of alternative transportation and parking, implementation challenges, lack of institutional and political support, and opposition from residents and drivers, opposition from some local retailers [39].

After conducting a literature review, it was found that transforming urban streets into car streets can negatively impact the environment. Specifically, it can increase traffic flow in surrounding areas, which leads to longer travel times and higher fuel consumption for drivers. Additionally, car users may have to spend more time searching for parking spaces on the city’s outskirts. Cavallo-Manzano, Lopez-Valpuesta, and Asencio-Flores [81] have also noted these negative consequences. Pedestrianized streets are exacerbated by the difficulty of pedestrians reaching their cars far from their locations [81] In the other vein, Murakami et al.’s study showed that pedestrian initiatives in the global competition will not lead to commercial revitalization or improvement in local retail streets around metro rail stations [40,56,91].

Pedestrians can comfortably walk in the city due to well-managed streetscape furniture, creating a walkable environment [56]. Pedestrianized streets are not recommended due to the difficulty of reaching vehicles [81] A recent study confirmed that converting urban streets exclusively for pedestrian use requires careful consideration of suitable design elements. These elements include sidewalks and suitable pavements, signage systems, aesthetics, and amenities [40,84]. They should be meticulously crafted to align with their intended purpose and elevate the pedestrian experience. The revised cases in which specific categories of vehicles allowed controlled access should be included, such as public buses and urban transportation, emergency services, delivery vans, and school buses [40,49].

In the other vein, studies suggest the need for traffic alternatives that consider the needs of all pedestrians and cyclists [55,92]. Murakami et al.’s study shows pedestrian initiatives in the global competition would lead to commercial revitalization or improvement in local retail streets around metro rail stations [40]. Recent research has suggested that pedestrianization initiatives may have little impact on revitalizing local commercial districts and improving retail streets. However, there are opportunities for pedestrianization in car-free zones in transit-oriented European cities, as highlighted by Murakami et al., which can contribute to local economic development [40]. On the other hand, Gonzalez-Urango et al. [82] and Brownrigg-Gleeson et al. [39] found that converting areas into pedestrian zones resulted in high citizen satisfaction, was perceived positively by businesspeople for commercial purposes and increased perceived attractiveness.

#### 4.1.4. Planning and Design Factors

This section examines how urban planning and design can encourage walking as a form of physical activity based on the existing literature.

- Seven planning and design factors characterize a pedestrianized street:
  1. Availability and accessibility enhance pedestrian connectivity and convenience. This factor can increase their movement speed and efficiency, reduce traffic congestion, promote transportation options like walking to make it more convenient for pedestrians, and positively impact their lifestyle and quality of life [85].
  2. Well-planned, well-designed pedestrian spaces create a more vibrant, inclusive, and economically prosperous community. Pedestrianization can be stimulated by increasing the number of stores, services, and companies, which provides an economic boost to the local area and creates job opportunities and wealth in the local economy [40], improving the shopping experience and thus attracting more customers to retailers, leading to increased sales volume; cafes and restaurants generate the highest revenues and enhance the volume of shopping and other commercial activities and ensure the financial viability of retail stores in inner cities that face increasing competition from suburban shopping malls [64].
  3. Design safe and comfortable sidewalks to prevent accidents like falls, slips, and collisions. A level surface with no sudden changes in elevation or unexpected obstacles allows individuals to navigate smoothly. This is particularly important for those with visual or cognitive impairments who may find moving around uneven or cluttered sidewalks challenging, for instance, diabetics who have foot ulcers and use mobility aids such as crutches. Investing in well-designed, barrier-free urban streets with accessible sidewalks can foster a healthier welfare community by creating more usable sidewalks [86].
  4. Provide a socializing, interactive, and enjoyable experience, leading to several positive outcomes, which can create more attractive urban streets that become popular destinations, attracting people from near and far to experience their unique atmosphere [41,49,87,90].
  5. Signage systems, barrier-free areas, streetscape furniture, public art, and pavements are crucial in urban planning and design. They enhance the pedestrian experience aesthetically [84] and significantly impact local economic development [40]. To achieve this, it is essential to thoughtfully arrange various street elements such as benches, bike racks, garbage cans, trees, shrubs, and grass. Proper placement of these elements can create a more comfortable and enjoyable environment for everyone.
  6. Integrating nature scores to gauge the number of green spaces in urban locales into pedestrian healthcare strategies can further enhance mental health. It is beneficial for individuals experiencing depression, bipolar disorder, anxiety, and urban stress, as it can promote well-being and healthy lifestyles [40].
  7. Management of green areas and linear green infrastructure is crucial in designing urban streets that combat air pollution, as stated by Jeong et al. in 2023 [44]. This factor highlights the importance of integrating nature into pedestrian healthcare strategies to promote well-being and mental health, as mentioned by Makram et al., in 2024 [40].
- Ten planning and design factors characterize a walkable street:
  1. Offer direct and easy street connectivity to different walkable activities in urban areas by planning and designing urban areas with sidewalk access, land-use density, compact design mixed-use, and diversity at a macro level [11,57,89] applying the natural-based approach of landscape fragmentation and habitat connectivity to pedestrian mobility planning, and offering pedestrian corridors, which helps pedestrians to be closer to moving freely across the city, and this could contribute to increasing the percentage of walking trips [57]. In addition to eliminating barriers or boundaries to pedestrian movements [92], consider the diversity factors of design scenario convenience [93] including micro-scale elements for comfort like sidewalk pavement quality and maintenance [86] street furniture, greenery, and trees [66] and blue spaces for preventing depression [94].
  2. Promoting local economic development is one of the strengths of walkable environments. According to a previous study, walkable environments have proven advantageous in fostering regional economic development. This is mainly because walkable communities tend to attract more foot traffic, increasing the visibility and accessibility of local businesses [66].
  3. Developing urban streets enables older adults to walk faster and with better balance and gait performance, making them more aesthetically connected to their neighborhood [66].
  4. Improving urban areas’ physical and functional organization to develop comfortable and safe walking paths and easily accessible opportunities for physical activity, social interaction, and health management can significantly improve urban walking experiences [24,41].
  5. Assessing the interaction of urban form related to the link between walkability and health, designers should propose expanding the implementation of green infrastructure [12,17].
  6. Enhancing the area’s appearance to reduce urban stress depends on the quality and effectiveness of infrastructure: sidewalk network width and condition [59] street crossings, connection to parking, on-street and off-street parking, tree canopy, building placement, restoration, and housing type and mix [62].
  7. Enhancing urban streets’ visual appeal and interest is essential to improve urban residents’ livability, public health, and well-being [89]. When designing these features, it is crucial to consider individual perspectives because what one person finds appealing may not be the same for others [95]. For instance, people’s perceptions of their surroundings can impact their behavior more than objective measurements [34].
  8. When designing a pedestrian network, six criteria are connectivity, linkage with other transportation modes, land use patterns, safety, path quality, and context [89]. Designers should consider urban design qualities, including imageability, enclosure, human scale, transparency, complexity [62,83], safety, conviviality, and vitality [70,79].
  9. Taking measures to reduce air pollution for low-income individuals who walk outdoors [96], such as managing green networks through urban street trees [44].
  10. Providing a pleasant urban climate experience alleviates the mental and emotional strain caused by high temperatures, solar radiation, and humidity. By promoting a balanced ecosystem and integrating green spaces and urban trees, we can effectively mitigate the detrimental effects of climate change on the environment and public health [97].

### 4.2. Storytelling Ideas: Episodic Narrative Interviews

At the onset of the first online video call, 30 individuals expressed their eagerness to tour the three districts of Cairo. Their objective was to observe and validate the numerous challenges presented by walking in these urban areas. Upon their return from the tour, they emphasized the importance of constructing streets that inspire physical activity as a healthy way of life for people with MetS. Moreover, they underlined the importance of prioritizing middle-class residents.

This section presents findings drawn from middle-class with metabolic syndrome Cairenes’ stories about walking as a physical activity in three regions of their city: Zamalek, downtown Cairo, and Heliopolis. It covers three main viewpoints. The first viewpoint discusses the experience of walking as a physical activity in the urban streets of Cairo. The second viewpoint relates to the types of urban streets they find most suitable for walking. The third viewpoint concerns the factors most important to them when choosing a walking route and the order in which they prioritize these factors.

#### 4.2.1. Is It Necessary to Have Urban Streets That Encourage Walking as a Physical Activity?

The results demonstrate that the participants reached a consensus on two crucial points that could help understand the pedestrian issue in the three regions under study. Firstly, all three areas were initially designed for walking. However, the rapid growth of urban development and the prevalence of automobile traffic over pedestrian traffic have made walking irrelevant in these areas. Secondly, none of the streets in the three regions can currently be classified as walkable. Only Old Cairo has a limited number of pedestrianized urban streets. Some participants shared their experiences in European and American cities where “pedestrianization” and “walkability” were available to help clarify their vision by drawing comparisons and bringing viewpoints closer together. The following passage recounts the experiences of two women in their sixties who walked along different pedestrianized streets. The first story was told on Hohe Straße, in Cologne, North Rhine-Westphalia, Germany. This bustling street, following Schildergasse, is the second-largest shopping district in the city. It is known for its affordable prices, historic architecture, and quiet atmosphere, as it is a pedestrianized and car-free street. Participants enjoy the numerous shops, cafes, and restaurants and stroll around that street.

A professor in her mid-sixties, a specialist in architecture living in Heliopolis, told her stories about the urgent necessity of such urban streets in Hohe Straße: “You cannot be on this street and not like to stroll; it has all the makings of the aesthetic appeal of an urban street. It was an enjoyment to walk along this street. It is attractive and eye-catching because of the historical buildings and landscape architectural features. [...] Unfortunately, this street is unsuitable for middle-class individuals with MetS. I tried to implement the exercise condition—cutting quickly for 30 continuous minutes to avoid hyperinsulinemia and inflammation, but the street was unsuitable”.

The second narrative was set on El Shawarbi Street, a popular spot for the middle class in downtown Cairo, Egypt, during the 1980s. Although it is still free of cars, the street is crowded with humans and street vendors, making it difficult for pedestrians to navigate. Additionally, the physical condition of the street has deteriorated, mainly in landscape architecture, such as street planting, materials, and furnishings.

A woman in her late sixties who works in fashion design and suffers from hyperinsulinemia and higher BMI told how she had witnessed the incident and mentioned the street’s designation for pedestrian use due to her living in Old Cairo. She stated: “The idea of designating certain streets for pedestrian use only was refreshing during that time. We were able to walk around without the constant presence of cars. However, we did not consider the need to walk for extended periods. Moreover, this street was not ideal for walking briskly or moving our arms freely”.

A pharmacist in his mid-seventies struggled with depression and found it challenging to interact with people and places. He shared that despite not liking walking, he learned about the health benefits of this physical activity and how it can combat depression. As a result, he decided to walk more often. He began planning daily walks around his home in El Zamalek and even ventured into other areas. [...] “Both experiences have shown me the importance of urban streets conducive to walking to stay physically active”.

A woman in her late sixties from El Zamalek who works administrative stated, "I have been experiencing sedentary behavior lately. Sitting in front of a computer screen all day leads to diabetic retinopathy and cataracts. I needed an easy opportunity to take a break and walk around. [...] However, my attempts to incorporate walking as a physical activity into my daily Cairo routine have failed. I cannot recall any streets in the three areas we are meeting today that would allow me to achieve this goal”.

#### 4.2.2. Which Urban Street Pattern Is More Suitable: Pedestrianized or Walkable?

The storytelling results highlighted three categories of opinions. The first category of the survey showed that eight participants prefer urban areas with walkable streets that allow for both pedestrians and cars. Meanwhile, four participants preferred pedestrianized urban streets that were easily accessible by car. The remaining 14 participants find that both patterns are appropriate, but factors that enable fast, comfortable, and safe walking activity to reduce the risk of metabolic syndrome still need to be considered.

An emeritus university professor at a public university in his 80s believes that both types of urban streets are necessary. He described his experience: “As a student, I strolled into Old Cairo and worked in New York City, USA. When I visited Times Square, I realized how wonderful it was to stroll around the city. I learned that New York City is the first walkable city in America. Times Square is the only pedestrian-only square in America. [...] However, due to my diabetic retinopathy and blurred vision, it became difficult for me to stroll around. I found walking around my neighborhood in El Zamalek helpful, but we need more urban streets where strolling is allowed as a physical activity without paying. These two types of urban streets can help people suffering from MetS symptoms to walk more comfortably”.

His wife, in her late eighties, suffering from diabetic Charcot neuropathy, worked as an emeritus professor at the same university. She said: “In my experience, we cannot classify any of the three areas in Cairo as walkable streets. Compared to the complete streets in New York City, we need ideas like this”. She suggested that Cairo invest in better infrastructure, such as better sidewalks and crosswalks, to make walking safer and more accessible. She also said the city needs to incentivize developers to create walkable communities.

A biochemistry teacher in his mid-seventies living in Heliopolis described a style of experience that did not exist in Cairo: “I have been to a completely new experience,” he said curiously. He also added, “[...] In Broadway, all means of transportation are present in one place and at one time. I was impressed by the harmony between all these activities. [...] I was amazed at the efficient public transportation system that allowed people to move quickly and easily around the city. I was also impressed by the variety of shops and restaurants and the diversity of people. I felt that the experience completely differed from what I was accustomed to in Cairo. Diabetes made me suffer from a fear of open and crowded traffic roads, which made me feel mentally or emotionally strained, frustrated, and nervous”.

#### 4.2.3. What Factors Should Be Added and in What Order?

This section offers insights into the narratives that underscore crucial factors to consider when planning and designing urban streets in Cairo’s three districts. These factors are especially pertinent to those with metabolic syndrome who wish to integrate walking into their daily regimen. The findings of this section focused on shedding light on insights that did not appear in the literature review as they relate to symptoms experienced by those with MetS. The factors have been classified into three categories: strategic, design-oriented, and technical.

- The First Group: Strategic Factors

The strategy for evaluating urban streets in Cairo for MetS mitigation was closely linked to the needs and interests of middle-class residents and the capabilities of local authorities. Thus, the main factors considered were the availability of urban streets, accessibility, and affordability. Below are some excerpts from narratives that highlight this issue.

An English language teacher in his mid-seventies, living in El Zamalek, who suffers from dispositional mindfulness and depression, stated: “I tried repeatedly to find this pattern when you contacted me to do this interview. Zamalek district has no pedestrian streets. I also searched for it in Heliopolis and many districts in Cairo. I found it only in Old Cairo, including downtown streets like El Shawarbi and El Alfi. […] Diabetes and irritable bowel made me confused and irritable. Walking relaxes me a little. I can no longer bear looking for streets to walk away from home. Making this type of urban street available is essential”.

A woman in her mid-sixties who works in the field of selling antiques in Heliopolis confirmed the words of a man. “The nature of my work makes me sit for long periods. I suffer from hammertoe arthropathy and weakness in my feet. My experience walking in the Heliopolis district indicates that streets designated for pedestrians do not exist. [...] In some old areas, you find arcade buildings where it is possible to walk in the arcade, but they are also not designated streets. As much as pedestrian walkable cities were developed in Heliopolis in 1905”.

A man in his late sixties who worked as a software engineer in information technology and had lived his entire life in Old Cairo said, “In the past, I used to drive my car to a major mall with outdoor activities and walk in the indoor streets. The place was wonderful but quite far from where I live. [...] Due to MetS, I suffer from dispositional mindfulness, which means that my ability to concentrate has declined, and my motor responses have also become less sharp. Because of this, I am looking for a place to stroll closer to my home. [...] needs to be a place I am familiar with to reduce the chance of getting lost”.

A man and a woman, both in their mid-seventies, working in the healthcare field in Heliopolis, shared their experiences. The man had dispositional mindfulness, while the woman had diabetes fatigue syndrome. A man stated, “I cannot drive because I always feel sleepy. It takes effort and time to get to the old Cairo hospital, and the cost of a taxi has doubled”.

After that, the woman added, “Indeed, Old Cairo has streets designated for pedestrians, and I thought several times about walking there. The outbound trip took an hour, and when I arrived, I could not walk; I felt tired from the slightest effort. [...] I have given up driving cars and prefer to walk near my home’s borders.

A man in his mid-sixties from Heliopolis shared his walking experience. He works at a low-paying job. Noting the current economic situation in the world, which has brought the middle class to the brink of poverty, he began his narration, “My doctor advised me to walk to relieve excess water from the various body systems and relieve the left kidney after I discovered I had metabolic syndrome. I realized I might suffer from kidney failure, as my father did. [...] Because I could not join private clubs or move into gated communities that I believed were walkable, walking urban streets became my only option”.

His wife, a pediatrician in her mid-sixties, mentioned, “Walking in one of the well-known parks in the district where I live is my habit. Since it became a gated park with high costs, I started walking along the park’s path. It embarrassed them as passers-by in buildings and urban streets could see them exercising and sitting on the edge of the garden wall for comfort. [...] I suffer from agoraphobia (or anxiety disorder), accompanied by a fear of hypoglycemia in a foreign environment. I avoid situations that might cause panic, feeling trapped, helpless, or embarrassed. [...] I believe that urban streets should be accessible to people in general and patients in particular, regardless of their economic and health conditions”.

- The Second Group: Design-Oriented Factors

According to the research findings, the current design or redesign of urban streets in the three districts prioritizes speed and efficiency over the health and well-being of pedestrians, including those with specific needs. The following display examines the factors pertinent to designing urban streets that encourage walking as a physical activity for the public, including middle-class individuals with MetS.

A woman in her mid-seventies from Old Cairo, a housewife, began her story, “I have diabetes fatigue syndrome; when I began adopting walking as a physical activity, I felt exhausted from walking too much. I was imagining that everything was fine. I was finding that my blood sugar levels were the same. [...] The doctor told me that walking Is a treatment with unique specifications; It must be continuous and fast, without stopping, for a specific distance ranging from 3000 to 10,000 steps, taking between half an hour and an hour. Here, I realized that factors must be considered in urban streets to make them suitable for those who want to reduce the risks of metabolic syndrome, such as providing paths that allow slow-paced and safe without stopping long hauls”.

A young woman in her mid-sixties, a resident of Old Cairo who works as an accountant in a company, suffering from high blood pressure and triglycerides, told the story, “One day, I was keen to try walking on one of the pedestrian streets in Old Cairo. I finished the path after 250 steps. I was accustomed to walking 5000 steps daily, so I had to walk it 12 times back and forth. Then I realized that the length of the street on which I walk does not exceed 150 m. [...] This is completely inconsistent with the requirements for walking as a physical activity”.

A veterinarian in his late sixties from El Zamalek, returning from Amsterdam with experience in walkable cities. He began his story by saying, “I have had metabolic syndrome for two years ago, with high blood pressure and type 2 diabetes. I need urban streets to walk every day, day and night, in all seasons of the year. […] The three districts in Cairo are challenging to classify as a walkable city. Amsterdam has been re-planned and designed to be walkable for days, not just 30 min. [...] Certainly, some areas of Cairo need to be developed to become walkable areas”.

A woman in her late sixties, a journalist living in El Zamalek, narrated, “I also suffer from many metabolic syndrome symptoms. […] I am interested in exploring opportunities to incorporate physical activity into my daily routine, and I am considering combining brisk walking with shopping. This would allow me to exercise while attending to necessary errands and potentially enhancing my shopping experience”. […] One challenge I faced in my brisk walking experience was combining shopping and walking as a physical activity in Heliopolis. The distance between home and the mall takes between 20 and 30 min at a moderate speed in unplanned and undesigned streets for this type of walking. [...] I found it different While trying to have the same experience in Berlin. [...]. Now, I know what about walkable cities, like Berlin, and I know about pedestrianized streets, such as the River Spree Walk, with a walking distance of 59 km, and the Lindenberg Corridor Walk, with a walking distance of 17 km”.

A woman in her late sixties who resides in Heliopolis and works as a principal in a girls’ school shared her experience regarding MetS. She suffers from Diabetic Charcot neuropathy, and she can feel the changes in her body. She said, “When I became aware of MetS, I started walking as a physical activity for more than half my life. However, the sidewalks in many areas must be more suitable for walking. The sidewalks should be free of obstructions and protrusions. […] While walking, I avoid hurdles on the sidewalks such as extension shops, kiosks selling newspapers, soft drinks, flowers, street vendors of all kinds, electricity rooms, and lampposts”.

A retired man in his seventies who lived in Heliopolis and worked as a researcher in agricultural sciences shared his experience, saying, “I have osteoporosis, which makes my bones weak and brittle. Due to this condition, I am at a higher risk of slipping and falling. I have tried changing my shoes multiple times to adapt to different walking conditions around my house. However, it has helped a little. [...] Safe walking and moving around can be difficult if the floor coverings are slick, slippery, or poorly maintained. Therefore, it is crucial to maintain the floor coverings in good condition and ensure they are safe to walk on to avoid potential hazards”.

A retired man in his mid-seventies who was suffering from osteoporosis, lived in Old Cairo, and worked as a legal consultant participated. “I tried looking at paving materials to pass the time. [...] One day, the surprise was that I avoided slipping more than twenty times due to going up and down from the sidewalk to the street until the end of my daily journey”.

A social researcher in her mid-seventies who lives in the same district narrated her story of mental and emotional stress, “Losing direction makes me panic. We must continue to focus on our targeted destination. [...] The features of the areas changed after they were transformed from an upscale residential neighborhood to an open commercial complex. [...] The urban chaos problem is repeated in the three districts, and its most essential features are congestion, street vendors, and visual and noise pollution. The biggest problem is that the culture of enjoying walking around has yet to be fully established despite the presence of historical buildings.

A man with diabetes fatigue syndrome in his mid-sixties who lives in Zamalek and works as a construction manager in a contracting company said, “I sometimes find myself in situations where I must take a break from walking and rest for a few minutes. This could mean sitting at a bus stop or on the sidewalk before continuing my journey. [...] Have you ever been in a situation where you tire of walking without finding a place to sit and relax? It is unfortunate when there are no seats available to take a break. That is why it is important to consider the benefits of having a place to rest and recharge while you are outside. Whether you need a quick break or want to enjoy the scenery, having a place to sit can make a big difference”.

Living in walkable neighborhoods may lead to reduced privacy due to increased population density and various walking infrastructures. A woman who suffered from brittle bones in her mid-sixties who lives in Zamalek and works as a researcher in earth sciences and geology shared her experience, stating, “I cannot walk quickly and move my arms freely because I fear accidentally colliding with someone. It has gotten so bad that I have started counting how many times I apologize for bumping into people, and it is often more than twenty times in a day”.

Another woman in her late sixties who has anxiety with a feeling of mental and emotional strain, who works as a university professor, said, “It is important to continue exercising regularly, but it can take time to stay motivated when things get monotonous. Boredom is almost killing me. After I decided to walk around the residential district, I live in in the Zamalek area, I felt anxious, frustrated, and lost passion. [...] The diversity of the experience of walking on an attractive street will be very motivating to continue any physical activity”.

- The Third Group: Technical Factors

An octogenarian industrial engineer from Heliopolis, who has an overactive bladder due to nerve damage, stated, “My stories of going to the bathroom are endless, as I have to enter a fast-food restaurant, or go down to subway stations, or return home from halfway. [...] Walking without public sanitary facilities is a matter of constant embarrassment, emptying the bladder”. Necessary and urgent, I did it once on one of the murals of a closed land that did not belong to anyone”.

Another woman in her late sixties who works in the field of theatre directing participated and shared her experience of brisk walking and the difficulties she faced in Heliopolis. She mentioned, "I used to struggle to enjoy my walking routine because it felt like another physical activity. However, incorporating some enjoyment into the routine could reduce my feeling rushed or overwhelmed. I learned to focus on and appreciate the scenery around me instead of just focusing on reaching my destination”.

An 80-year-old mathematics teacher residing in Old Cairo said, “My experience as a person with diabetic retinopathy and cataracts. Direct sunlight and distorted lighting at night increase the risk of blurred vision [...]. I remember that this used to cause me many problems that made me lose my direction and unable to enjoy a quick and quiet walk simultaneously”.

A man in his mid-seventies who lives in Old Cairo and works in a public bookstore said, “I have been experiencing high blood pressure while walking and initially thought it was due to physical activity. However, my doctor informed me that the real cause is the air pollution caused by car exhaust emissions. […] The air pollution causes arteries to harden, and fat cells accumulate inside them, affecting the heart muscles”.

A woman in her late seventies who lives in Heliopolis and is a housewife told the story, “I have a contradictory experience where high temperatures can increase the risk of irregular blood glucose levels, but at the same time, it can reduce high blood pressure. Climate extremes can also affect metabolic syndrome. […] Climate treatments are designed by specialists who understand how to manage these effects”.

## 5. Discussion: Exploring and Prioritizing Factors

This study acknowledges the importance of walkable communities and how they benefit all residents, promoting healthy behaviors such as walking. Additionally, we can emphasize that designing communities to mitigate MetS risks inherently fosters an environment accommodating everyone’s needs, contributing to overall health and well-being.

There has been much discussion regarding pedestrianized and walkable urban streets in recent years. However, studies have yet to be conducted to gauge people’s preferences between these two walking styles, specifically for physical activity. Furthermore, further research is needed to determine which of these styles is best suited to reduce symptoms of MetS. This study could provide valuable insights into planning and designing urban streets for physical activity. It will also help inform policymakers on how to create urban streets for pedestrians while also considering the needs of the middle class with MetS.

This study offers a comprehensive framework for urban street planning and design aimed at prioritizing walking as a physical activity among middle-class individuals affected by metabolic syndrome. Conducted through a scoping review, the research compares pedestrianized and walkable streets while identifying symptoms associated with metabolic syndrome. Employing storytelling techniques, the study examines three areas of Cairo, utilizing open-source data to analyze the factors influencing urban street planning and design, their prioritization, and their relationship with metabolic syndrome symptoms. The ultimate objective is to propose potential solutions to mitigate these symptoms effectively.

After conducting a storytelling study and gathering participants’ opinions, the researchers analyzed the data to discover and prioritize the most significant factors for the participants. This analysis identified three distinct groups and 15 factors ranked based on their perceived importance (Table A1B in Supplementary Materials). Concerning middle-class individuals with MetS, despite the participants’ age variation, all agreed that these factors are the most critical, and their order is as stated in Table 3.

### 5.1. Comparison with Prior Studies

In line with previous studies, the fundamental difference between the terms “pedestrianized” and “walkable” urban streets is that the former eliminates any trace of motorized traffic in the pedestrian area. At the same time, the latter facilitates pedestrian accessibility by integrating the motorized traffic network with pedestrian traffic paths. So, the first term relies on pedestrian paths in absolute terms. In contrast, the second encourages people to walk on foot with automated transportation.

The content analysis results confirmed that pedestrianized and walkable urban street patterns should consider planning and design factors that encourage walking from a ‘public health’ and ‘smart growth’ perspective. Other studies have also reported its importance for well-being, sustainable development, climate change, tourism [97] and the local economy [39].

Research has shown that walking as a physical activity can reduce the risk of chronic diseases, improve cardiovascular health, prevent type 2 diabetes, enhance mental health, and maintain physical function [24,37,43]. However, more in-depth research is necessary in urban planning and design to examine how MetS symptoms impact urban street planning and design. Comparing Cairene’s novels to previously published research has shed light on some noteworthy observations in this regard.

Ideas related to storytelling have revealed that walking as a physical activity is strongly linked to strategic factors that involve the availability of pedestrianized and walkable streets in urban environments. It is essential to enhance accessibility and reach these streets quickly and directly, as they are considered destinations. In addition, the participants highlighted that older adults with better walking speed, balance, and gait performance are perceived to be more in tune with their surroundings. These ideas are consistent with many studies by Lui [65] and De Vos et al. [66] on walkability in the neighborhood environment, walking time, and functional mobility in older adults. Participants also emphasized that urban streets should be available at the lowest cost, which aligns with Murakami et al. [40] about enhancing local economic development by saving money and reducing private transportation. Then, to provide greater access to essential services via walking, the availability of street patterns in each residential district should be approached with this economic logic that will help the middle classes.

Second, urban streets should include pedestrian paths and automated networks to provide a comprehensive and user-friendly experience. This environment allows pedestrians to interact with their surroundings more efficiently without interference from motorized traffic. Storytelling of design-guided factors consistent with what has been reported in many studies on walking as a physical activity in a safe and supportive environment has been very beneficial. For example, Wei et al. [11] reported the dominant influence of compact design for land use on pedestrian behavior. Gonzalez-Urango [82] stated the effect of involving multiple stakeholders and planning and design criteria. Gregg [82] noted the impact of diversity and novelty of daily activities on enhancing the vitality and attractiveness of a neighborhood, and Erturan and Spek [93] considered physical and perceptual factors through the diversity of design scenarios.

Likewise, Delso et al. [57] and Lee and Kim [92] pointed out that continuity of paths, pavement availability, and removal of physical barriers can make walking more accessible by creating a clear boundary between pedestrian and vehicle areas. Lui and Wong [65] mentioned that an environment that provides walking at a faster pace and with better coordination achieves continuity of movement quickly and efficiently and protects against slipping and falling risks. Cysek-Pawlak and Pabich [52] and Li et al. [95] focused on the associations between visual features of the built street environment that make walking attractive. According to the narratives of Cairo locals, having a pleasant walking experience and reducing fatigue is possible by providing enough activities along the streets. Zainol et al. [84] revealed that people’s satisfaction levels decreased significantly due to the lack of aesthetics and amenities that promote a walkable environment.

Third, many of the ideas presented in Cairenes’ narratives highlight the need for technical factors for better urban street planning and design to encourage walking as a physical activity in urban areas, emphasizing the adequacy of facilities and reducing photosensitivity. Reducing sensitivity to light is essential for those with eye diseases, and using air pollution and controlling the microclimate is necessary for those with inflammation and blood vessel damage. These ideas are consistent with innovative growth principles emphasizing the importance of healthy streets and environmental performance measures. The results conducted by Yu et al. [96] and Jeong et al. [44] suggested green networks for reducing air pollution, while Velázquez et al. [97] recommended controlling extreme climatic conditions by incorporating vegetation areas with trees to minimize sun exposure. Kent et al. [12] considered the interaction of urban form-related walkability and health interaction, while Shartova et al. [17] proposed expanding green infrastructure and urban form implementation.

### 5.2. Policy and Planning Implications

According to research, walking can positively affect citizens’ health from a public health and smart growth perspective. The findings suggest that decision-makers should improve urban street planning and design factors to encourage physical activity for middle-class individuals with MetS. These results have significant implications for policymakers promoting physical activity among citizens. When planning and designing urban streets for middle-class individuals with MetS in Cairo, there are 15 factors to consider, grouped into three categories as mentioned in Table 2 above, as follows:

- The first group aims to strategically plan urban streets near residential areas to ensure easy and free access for all. It includes three crucial strategic factors:
  1. “Availability” is more than simply being present; it encompasses having urban streets conveniently near residential areas. The planning and design strategy for this factor is to ensure that urban streets can be accessed on foot without requiring any transportation and that people can return to their starting point without feeling exhausted, which is an essential consideration for urban planners and designers as a priority.
  2. “Ease of access and use” refers to the need for better access to urban streets conducive to walking as a form of physical activity. This factor aims to create streets that are easily accessible, safe, and free from physical barriers, allowing people to enter them spontaneously and without hesitation. The goal is to encourage people to walk more by providing streets suitable for walking at any time.
  3. The concept of “affordability” aims to allow people to engage in physical activities on city streets without limitations. This factor considers the initial expenses, ongoing maintenance costs, and long-term affordability for different socioeconomic groups. Local authorities must encourage businesses to invest in infrastructure promoting physical activity by offering tax credits and subsidies, land-use changes, and store density, mainly cafes, restaurants, and non-tradable local consumption activities.
- The second group pertains to design-oriented factors, focusing on urban street performance and quality. These factors ensure that streets are compatible with their intended purpose. During the discussion, participants agreed that to make an urban street suitable for physical activity, such as walking for middle-class individuals with MetS, the following factors should be prioritized in order:
  1. “Continuity of movement” is the concept of creating urban streets that allow people to walk long distances quickly and efficiently within a designated time and distance following medical guidelines.
  2. “Combat fatigue effectively” by embracing a regular walking routine and ensuring adequate rest areas are available. This factor can help walkers improve their energy levels and overall well-being by prioritizing resting points regularly during their walking sessions and events.
  3. “Eliminating obstacles and interference” that pedestrians face can help to consolidate their path and avoid potential hazards while walking. This factor emphasizes the importance of looking forward instead of downward while walking. It is essential to consider whether the street can accommodate street furniture, electrical poles, street vendors, or other occupations that could impede pedestrian movement.
  4. “Maintain floor coverings” in excellent and safe condition to prevent potential hazards from slippery or rough floor materials. This factor depends on a proactive approach to maintaining floor coverings, which will help prevent accidents and promote a safe environment for everyone who walks on your floors. Regularly inspect, clean, and repair any damage or defects without delay.
  5. “Stabilizing the urban form without significant changes” to its immediate surroundings, such as excessive development or improvement. This factor contributes to reducing anxiety resulting from the fear of getting lost or disoriented. It also helps prevent health issues such as high blood sugar and pressure levels, which can lead to symptoms such as blurred vision and confusion. This issue is especially relevant for those who suffer from forgetfulness or mental confusion due to aging or aging-related diseases.
  6. Providing enough “personal space” for pedestrians to stretch while walking can be highly beneficial, particularly for individuals who have to sit for long periods. It allows them to engage in physical activity while going about their day. The availability of personal space to stretch will enable pedestrians to loosen their muscles, release tension, reduce psychological pressure and anxiety, and alleviate boredom.
  7. Offering suitable “seating areas” for practicing stretching exercises while sitting. This factor can significantly improve pedestrians’ mental health, mood, and cognitive function. By providing such spaces, individuals will have access to a place where they can alleviate symptoms of anxiety, depression, stress, and sadness through exercise.
- The third group pertains to technical factors determining whether the street suits its intended purpose. It also considers whether it is ideal for middle-class individuals with MetS. This group comprises four pertinent land-use factors: adequacy of facilities, various mixed-use activities, illumination, pollution, and climate. These factors are listed in order of priority as follows:
  1. “Adequate restroom facilities” near pedestrian areas can improve accessibility and safety and prevent bladder issues.
  2. Using lighting units compatible with morning and evening hours is recommended to minimize eye diseases and light sensitivity. The illumination should be adjusted to the time of day to prevent discomfort. Investing in adjustable and natural light is advisable, and position lighting fixtures appropriately to avoid glare and shadows. These measures can help maintain optimal eye health, leading to an overall improvement in well-being.
  3. Protection from air pollution and climate extremes to avoid increased inflammation and blood vessel damage.
  4. Provide visually enjoyable and aesthetically pleasing views to enhance pedestrian urban streets’ user experience. When navigating an unfamiliar location, the fear of getting lost can be a source of anxiety. However, this anxiety can be significantly reduced by incorporating pleasing features like natural scenery, artwork, or architectural elements. Creating a functional and beautiful environment allows users to feel more comfortable while exploring their surroundings.
  5. Combining brisk walking with shopping by providing different mixed-use activities with diverse design scenarios is convenient to provide opportunities for outdoor activities and events. It helps pedestrians exercise while attending to necessary errands and enhances my shopping experience to enhance an individual’s mood.
  6. Develop attractive destinations for individuals with metabolic syndrome, leading toward pedestrian corridors that meet prevention measures.

### 5.3. Limitations Studies and Future Research

A strength of the current study lies in eliciting a range of objective insights with a sample of participants targeted in three spatially differentiated urban districts after filtering them from a larger sample, which enabled the examination of objective and perceived environmental measures across a range of three factors, strategic, design-oriented, and technical and compared them to results from peer studies that measured objectively and published internationally.

However, future studies should address the current study’s limitations by recruiting participants from various socio-economic backgrounds, i.e., including low and high-income groups, in the future. A larger sample size should also be surveyed to capture population diversity better. In addition, alternative data collection and analysis research methods and techniques should be explored to capture better different population groups’ views, such as web polls, which provide a sample of countries with similarities to Egypt’s current circumstances from the Global South. 

Public health literature suggests that walking is effective for combating MetS symptoms. However, it is still being determined to what degree urban streets should be intentionally planned and designed to encourage walking among middle-class individuals. This question requires professionals from two fields: public health and smart growth. Public health researchers with expertise in metabolic syndrome can provide insight into the specific symptoms to be considered in street planning and design. Meanwhile, urban street planning and design practitioners specializing in smart growth can ensure that streets are designed to encourage walking and other physical activities. By leveraging the knowledge and expertise of both fields, we can create urban streets that combat metabolic syndrome and improve middle-class individuals’ overall health and well-being.

### 5.4. Deductive Arguments and Conceptual Framework

Planning and designing streets to encourage physical activity, focusing on their prioritization, should consider public health and smart growth prerequisites. The proposed framework aims to enhance urban street planning and design to meet the physical activity requirements of middle-class individuals with MetS and help them make informed lifestyle choices. 

A conceptual framework includes four phases. The first phase extracts the main commonalities, fundamental differences, strengths (facilities), weaknesses (barriers) between pedestrianized and walkable urban streets, and planning and design factors for each street. The second phase is focused on exploring the symptoms of MetS and prevention measures. The third phase is focused on enhancing urban street planning and design by integrating effective solutions to alleviate the symptoms of metabolic syndrome. This phase was carried out by considering all relevant factors from existing literature while prioritizing the viewpoints of a chosen group of residents from three districts in Cairo, Egypt. The last phase presents an action plan to counteract the symptoms of MetS through planning and designing urban streets, focusing on walking as a physical activity. Practitioners and policymakers should consider urban street planning and design factors to mitigate symptoms of MetS syndrome. This procedure can be achieved through an action plan consisting of five steps. Conduct a comprehensive analysis of the definition and impacts of MetS symptoms based on a medical literature review. 

This study delves into an in-depth analysis of MetS symptoms, exploring its definition and impacts through a comprehensive review of medical literature. It further examines various strategies employed in urban planning and design to mitigate MetS, focusing particularly on urban streets in both Global North and South communities. Through a blend of theoretical exploration and practical insights, the study identifies design-oriented approaches, technical factors, and potential challenges associated with addressing MetS. An experimental project is devised to solicit feedback and refine mitigation strategies, with local experts’ input crucial in evaluating these approaches’ benefits and drawbacks.

**Figure 2:**
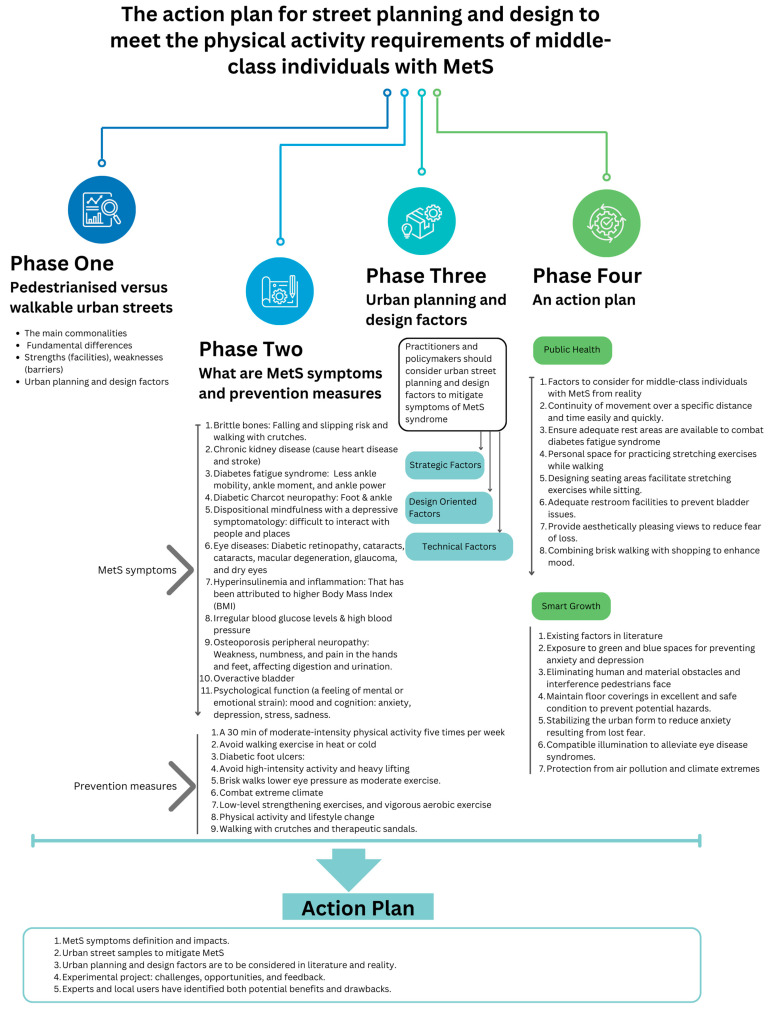
The action plan.

Figure 2 illustrates the action plans for street planning and design to meet the physical activity requirements of middle-class individuals with MetS, including strategic, oriented design and technical factors addressing ‘public health’ and ‘smart growth’ challenges. The conceptual framework provides a comprehensive and practical approach for researchers and practitioners to rank the critical urban street planning and design factors that promote physical activity while ensuring ‘public health’ and ‘smart growth’ challenges. The framework is designed to consider multiple variables, including strategy, the availability and accessibility of these streets easily and quickly, and the affordability of use. Design-oriented factors include keeping things moving quickly and easily: distance and time, removing obstacles to looking ahead, preventing the risk of slipping and falling, avoiding stress resulting from the fear of losing direction and getting lost, providing personal spaces for motor stretching exercises, and comfortable sitting for stretching exercises from a seated position, and enjoying the walking routine by reducing feeling tired. The technical factors include adequate facilities and a variety of uses and activities as an incentive, reducing light sensitivity, and controlling climate fluctuations and air pollution.

Moreover, practitioners and policymakers can use this framework to assess the effectiveness of their initiatives, including implementing urban street design improvements, such as bike lanes, public transportation, and pedestrian-friendly infrastructure. This framework has broad applicability and can be used in various contexts, from urban centers to suburban neighborhoods, to evaluate the impact of urban design on public health and physical activity. Overall, this framework is valuable for researchers, practitioners, and policymakers alike, helping to create healthier, more active, and more livable communities for all.

## 6. Conclusions

This study presents a comprehensive conceptual framework for urban planners and designers to mitigate metabolic syndrome risks and socioeconomic disparities among middle-income populations. The framework contributes to refining urban street planning and design, explicitly promoting walking to alleviate metabolic syndrome symptoms in urban areas. The framework addresses three key aspects: firstly, assessing the urgency for planning and designing urban streets that facilitate pedestrian activity; secondly, determining the most suitable type of urban street for individuals with MetS—whether a “pedestrianized” or “walkable” street; and thirdly, prioritizing urban street planning and design factors that emphasize walking as a physical activity.

Scoping review analyses showed that pedestrianized and walkable urban streets have many planning and design factors in common. However, they have different goals. The review results highlighted each type’s commonalities, key differences, strengths (facilities), weaknesses (barriers), and planning and design factors.

We conducted a study investigating the planning and design considerations for two kinds of urban streets that promote pedestrian physical activity—“pedestrianized” or “walkable”. The case study explores how urban planning and design can promote walking to combat MetS among middle-class patients in major cities of the Global South, particularly Cairo, Egypt. This study utilizes storytelling techniques to identify the most appropriate urban streets for middle-class individuals with MetS in Cairo, Egypt. This study aims to help develop plans and urban paradigms that promote walking as a physical activity on Cairo streets. The analysis revealed that Cairenes are highly receptive to car-free pedestrian paths that make it easier for pedestrians to move around the city. Additionally, middle-class individuals who suffer from MetS in Cairo strongly agree with these findings and emphasize the importance of designing these walking paths to meet their specific needs, as determined by doctors, to address metabolic MetS.

This study prioritizes urban streets based on public health and smart growth criteria, categorizing factors into three groups: strategic, design-oriented, and technical. Strategic factors include accessibility, affordability, and availability. Design-oriented factors focus on facilitating movement, ensuring safety, and enhancing the overall walking experience. Technical factors address specific health considerations such as facilities for overactive bladder, reducing light sensitivity, and controlling climate extremes to mitigate health risks associated with metabolic syndrome. 

Despite extensive research on planning pedestrian streets in Cairo, Egypt, a comprehensive synthesis is needed to effectively address measures for promoting physical activity and mitigating metabolic syndrome among the middle class. Future research should prioritize the development of precise design guidelines and implementation strategies tailored to these challenges. A thorough examination of the specific hurdles in planning and designing urban streets to promote walking, particularly among middle-class cohorts, is essential. Decision-makers must prioritize creating pedestrian-friendly streets while addressing strategic, design-oriented, and technical considerations. Methodical data collection incorporating people’s preferences is crucial for devising practical strategies. Subsequent investigations should explore the interplay between public health imperatives and intelligent urban growth, enabling medical professionals to effectively tailor interventions to local contexts and address metabolic syndrome symptoms.

## Figures and Tables

**Table 1:** The results of materials yielded and their categories through the subject area, categories, and journal characteristics, including article titles, h-index, and journal quartiles.

SA *	Environmental Science			Social Science					
C **	Public Health, Psychology, and Nature Landscape Planning			Geography, Planning, Development, and Urban Studies			Transportation		
JC ***	AT ^+^	*h*-index	Q ^#^	ST ^€^	*h*-index	Q	ST	*h*-index	Q
	*Ageing International*	41	3	*Cities*	114	1	*European Transport*	23	3
	*American Journal of Preventive Medicine*	241	1	*Computers, Environment, and Urban Systems*	105	1	*Journal of Traffic and Transportation Engineering*	40	1
	*Annals of Behavioral Medicine*	145	1	*Journal of Urbanism*	27	2	*Transport Reviews*	100	1
	*International Journal of Environmental Research and Public Health*	167	2	*Journal of Urban Design*	54	1	*Transportation Research Part A: Policy and Practice*	153	1
	*Land*	42	2	*Journal of Urban Planning and Development*	51	2	*Transportation Research Procedia*	59	0
				*Habitat International*	102	1			
				*Health and Place*	129	1			
				*IATSS Research*	36	1			
				*Land Use Policy*	138	1			
				*Open House International*	15	1			
				*Social Sciences*	35	2			
				*Sustainable Cities and Society*	103	1			
				*Sustainability*	136	1			
				*Procedia—Social and Behavioral Sciences*				67	0
				*Transport Policy*				113	1
	*Journal of Transport and Health*							46	1

^+^ AT—Article title, * SA—Subject areas, ** C—Categories, *** JC = Journal characteristics including, ^€^ ST—Source title, and ^#^ Q—journal quartile.

**Table 2:** Three age groups were analyzed for 30 participant characteristics: age, gender, number of participants, job specialization, residence place, and MetS symptoms.

Age Group		Gender	Serial	Job Specialization	Residence Place	MetS Symptoms
The sixties	Mid-sixties	Woman	1	A specialist in architecture	Heliopolis	Hyperinsulinemia and inflammation
	The late sixties	Woman	2	Fashion design	Old Cairo	Hyperinsulinemia and higher BMI
	The late sixties	Woman	3	Administrative job	El Zamalek	Diabetic Charcot neuropathy
	Mid-sixties	Woman	4	Selling antiques	Heliopolis	Hammertoe arthropathy
	The late sixties	Man	5	A software engineer in the field of information technology	Old Cairo	Dispositional mindfulness
	Mid-sixties	Man	6	A low-paying job	Heliopolis	Chronic kidney disease
	Mid-sixties	Woman	7	A pediatrician	Heliopolis	Anxiety: a feeling of mental or emotional strain
	Mid-sixties	Woman	9	An accountant	Old Cairo	High blood pressure and triglyceride
	The late sixties	Man	8	Veterinarian	El Zamalek	High blood pressure and type 2 diabetes
	The late sixties	Woman	10	A journalist	El Zamalek	Many metabolic syndrome symptoms
	The late sixties	Woman	11	A girls’ school principal	Heliopolis	Diabetic Charcot neuropathy: Foot and ankle
	Mid-sixties	Man	12	Construction manager	El Zamalek	Diabetes fatigue syndrome
	Mid-sixties	Woman	13	A researcher in earth sciences and geology	El Zamalek	Brittle bones
	The late sixties	Woman	14	A university professor	El Zamalek	Anxiety: a feeling of mental or emotional strain
	The late sixties	Woman	15	Theatre directing field	Heliopolis	Anxiety: a feeling of mental or emotional strain
The seventies	Mid-seventies	Man	16	A pharmacist	El Zamalek	Depression and finding it difficult to interact with people and places
	Mid-seventies	Man	17	A biochemistry teacher	Heliopolis	A feeling of mental or emotional strain with frustration and nervousness
	The late seventies	Man	18	An English language teacher	El Zamalek	Dispositional mindfulness and depression
	Mid-seventies	Man	19	In the healthcare field	Heliopolis	Dispositional mindfulness
	Mid-seventies	Woman	20	In the healthcare field	Heliopolis	Diabetes fatigue syndrome
	Mid-seventies	Woman	21	A housewife	Old Cairo	Diabetes fatigue syndrome
	The late seventies	Man	22	A researcher in agricultural sciences	Heliopolis	Osteoporosis peripheral neuropathy
	Mid-seventies	Man	23	A legal consultant	Old Cairo	Osteoporosis peripheral neuropathy
	Mid-seventies	Man	24	A social researcher	Old Cairo	A feeling of mental or emotional strain
	Mid-seventies	Man	25	A public bookstore	Old Cairo	A high blood pressure
	The late seventies	Woman	26	A housewife	Heliopolis	High blood glucose and pressure
Octogenarian (between 80 and 89 years old)		Man	27	Emeritus university professor	El Zamalek	Diabetic Charcot neuropathy: Foot and ankle
		Woman	28	Emeritus university professor	El Zamalek	Hammertoe arthropathy
		Man	29	An industrial engineer	Heliopolis	Overactive bladder
		Man	30	A mathematics teacher	Old Cairo	Diabetic retinopathy

**Table 3:** Sixteen factors in three groups for planning and designing urban streets for middle-class individuals with MetS.

Strategic Factors	Design-Oriented Factors	Technical Factors
1. Availability: Conveniently located near residential areas, with easy accessibility without transportation.	4. Continuity of movement over a specific distance and time easily and quickly.	11. Adequate restroom facilities to prevent bladder issues.
2. Accessibility: Ensure no physical barriers hinder direct, safe, and quick entry for easy accessibility.	5. Combat fatigue effectively by ensuring adequate rest areas are available.	12. Compatible illumination to alleviate eye disease syndromes.
3. Affordability: Accessible urban streets to everyone without cost.	6. Eliminating human and material obstacles and interference pedestrians face: Providing the ability to look forward instead of down at them.	13. Protection from air pollution and climate extremes to avoid increased inflammation and blood vessel damage.
	7. Maintain floor coverings in excellent and safe condition to prevent potential hazards.	14. Provide aesthetically pleasing views to reduce fear of loss.
	8. Stabilizing the urban form to reduce anxiety resulting from lost fear.	15. Combining brisk walking with shopping to enhance mood.
	9. Providing personal space for pedestrians to practice stretching exercises while walking.	16. Developing attractive destinations for pedestrian flow.
	10. Designing seating areas facilitates stretching exercises while sitting.	

## Data Availability

The data presented in this study are openly available in FigShare at https://figshare.com/articles/dataset/Data_Metabolic_Syndrome_in_Cairo_Egypt/25195889/1 (accessed on 5 February 2024).

## Supplementary Materials

The following link shows the appendices, which can be downloaded at: https://doi.org/10.6084/m9.figshare.25195889.v2. Table A1A: A Summary of 38 Selected Peer-Reviewed Articles in 26 Journals Was Published from 2005 to 2024; Table A1B. Thirty Participants Ranked Three Groups and Sixteen Factors Based on Perceived Importance.
